# 568. Comparison of Humoral Immune Response to the SARS-CoV-2 BNT162b2 Vaccine Between Solid Organ Transplant Recipients and Healthy Controls

**DOI:** 10.1093/ofid/ofab466.766

**Published:** 2021-12-04

**Authors:** Ahmad Yanis, Zaid Haddadin, Andrew Speaker, Danya Waqfi, Rana Talj, Danielle A Rankin, Lauren Ezzell, Marcia Blair, Joan Eason, Rebekkah Varjabedian, Lora Thomas, James Chappell, Natasha B Halasa, Natasha B Halasa

**Affiliations:** 1 Vanderbilt University Medical Center, Nashvill, Tennessee; 2 Vanderbilt University Medical Center; Division of Pediatric Infectious Diseases, Nashville, Tennessee

## Abstract

**Background:**

Severe acute respiratory syndrome coronavirus 2 (SARS-CoV-2) is associated with increased morbidity and mortality in immunocompromised individuals, including solid organ transplant recipients (SOTR). Despite being excluded from phase 1-3 SARS-CoV-2 vaccine clinical trials, SOTR were identified as high-risk populations and prioritized for vaccination in public health guidelines. We aimed to evaluate the antibody response to two doses of the BNT162b2 (Pfizer-BioNTech) vaccine in SOTR as compared to healthy controls (HC).

**Methods:**

SOTR and HC scheduled to receive two doses of BNT162b2 vaccine and able to complete required follow-up visits were enrolled. Blood specimens were collected from participants before receiving the first and second doses and 21-42 days after the second dose. Enzyme-linked immunosorbent assay (ELISA) was used to detect immunoglobulin G (IgG) to the SARS-CoV-2 spike receptor-binding domain (RBD). Generalized estimating equations with a working independence correlation structure were used to compare anti-RBD IgG levels between SOTR and HC at each study visit and within each group over time. All models were adjusted for age, sex, and pre-vaccination seroreactivity in the ELISA.

**Results:**

A total of 54 SOTR and 26 HC were enrolled, with mean (SD) ages of 72 (3.6) and 62 (6.7) years, 61% and 35% were male, and 91% and 88% were white, respectively. The most common organ transplant types were kidney (41%) and liver (37%). All SOTR were receiving calcineurin inhibitors. The median time post-transplantation was 7 years. SOTR had markedly lower mean anti-RBD IgG levels when compared to HC with adjusted mean differences of -0.76 (95%CI: [-1.04, -0.47]; p < 0.001) ELISA units (EU) and -1.35 (95%CI [-1.68, -1.01]; p < 0.001) EU after the first and second doses, respectively (Figure 1). Both groups had a significant increase in anti-SARS-CoV-2 IgG levels after the second dose. However, the magnitude was lower in SOTR, 0.49 (95%CI [0.31, 0.69]; p < 0.001) EU than in HCs, 1.08 (95% CI [0.91, 1.24]; p < 0.001) EU.

Figure 1.

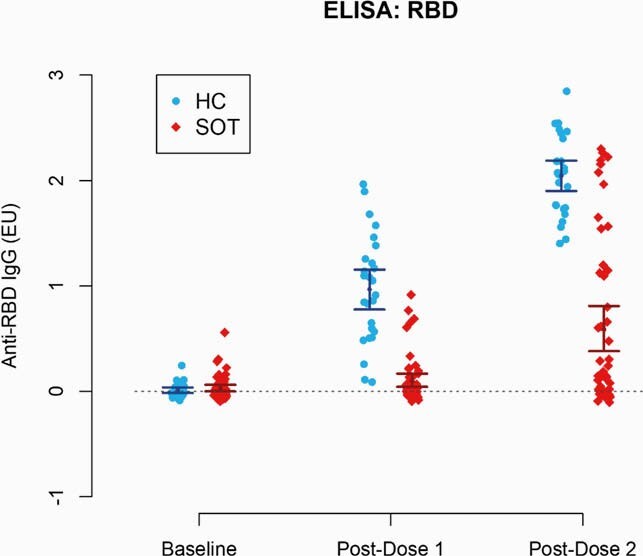

Anti-SARS-CoV-2 RBD IgG levels in solid organ transplant recipients and healthy controls before receiving the BNT162b2 vaccine (baseline), post-vaccine dose 1, and post-vaccine dose 2.

**Conclusion:**

Our study showed SOTR mounted weaker humoral immune responses than HC to SARS-CoV-2 vaccines. Given a lower response, SOTR should continue to practice social distancing and masking until data on vaccine efficacy are available in this vulnerable population.

**Disclosures:**

**Natasha B. Halasa, MD, MPH**, **Genentech** (Other Financial or Material Support, I receive an honorarium for lectures - it's a education grant, supported by genetech)**Quidel** (Grant/Research Support, Other Financial or Material Support, Donation of supplies/kits)**Sanofi** (Grant/Research Support, Other Financial or Material Support, HAI/NAI testing) **Natasha B. Halasa, MD, MPH**, Genentech (Individual(s) Involved: Self): I receive an honorarium for lectures - it's a education grant, supported by genetech, Other Financial or Material Support, Other Financial or Material Support; Sanofi (Individual(s) Involved: Self): Grant/Research Support, Research Grant or Support

